# Differences in Salinity Stress Responses Across Developmental Stages and Tissue Regions in *Saccharina japonica*

**DOI:** 10.3390/ijms27135910

**Published:** 2026-06-30

**Authors:** Wen Lin, Jiexin Cui, Jincheng Yuan, Tao Liu

**Affiliations:** 1 College of Fisheries, Jimei University, Xiamen 361021, China; 202311710010@jmu.edu.cn; 2 State Key Laboratory of Marine Environmental Science, College of Ocean and Earth Sciences, Xiamen University, Xiamen 361102, China; yuanjincheng@stu.xmu.edu.cn; 3 Southern Marine Science and Engineering Guangdong Laboratory, Zhuhai 519000, China

**Keywords:** *Saccharina japonica*, salinity stress, developmental stage, tissue region, antioxidant defense, osmotic regulation

## Abstract

*Saccharina japonica* is an economically important stenohaline brown seaweed whose growth and yield are significantly affected by frequent salinity fluctuations in coastal aquaculture areas. The differences in salt tolerance and response characteristics among developmental stages and among tissue regions of adult-stage thalli remain unclear, and the dynamic temporal patterns of responses across stages and tissues have not been systematically elucidated. In this study, we compared the physiological responses of juvenile and adult-stage thalli under varying salinity conditions and further analyzed the responses of the basal, middle, and tip regions of adult-stage thalli to define stage- and tissue-specific patterns of salt tolerance. The results indicate that low-salinity stress caused more severe injury than high-salinity stress, as reflected by sustained decreases in Fv/Fm, increased accumulation of MDA, and aggravated tissue decay with green-rot symptoms. Juvenile sporophytes exhibited higher salt tolerance than adult-stage thalli, and within the latter, tolerance differed markedly among tissue regions, with the basal region showing greater tolerance than the middle and tip regions. The basal region maintained higher photosynthetic activity, lower lipid peroxidation levels, and more stable antioxidant and osmotic regulatory responses under stress, whereas the tip region experienced early photosynthetic inactivation and irreversible damage. qRT-PCR results showed that antioxidant- and osmotic-regulation-related genes, including *SjGSH*, *SjGST*, *SjPro*, *SjSOD*, and *SjPOD*, were differentially expressed under salinity treatments at 24 h and 72 h, and their expression dynamics were generally consistent with the changes in physiological indicators. Overall, this study demonstrates that the response of *S. japonica* to salinity stress exhibits clear developmental stage-dependent differences and tissue-specific characteristics. In adult-stage thalli, the tip region may serve as a sensitive monitoring region for low-salinity damage, the middle region may serve as a transitional region for evaluating the progression of stress-induced damage, and the basal region may be an important region for maintaining thallus growth and physiological homeostasis. This study also provides experimental evidence for low-salinity stress risk assessment, the management of key growth stages, the monitoring of sensitive tissues, and the evaluation of salt tolerance traits during *S. japonica* aquaculture.

## 1. Introduction

*Saccharina japonica* is a large, economically important brown seaweed belonging to the phylum Ochrophyta, class Phaeophyceae, order Laminariales, family Laminariaceae, and genus *Laminaria*. It is a key macroalga along the North Pacific coasts, contributing significantly to coastal economic development, nearshore ecological restoration, and nutrient remediation [1,2]. The *S. japonica* industry in China plays a prominent role in both global and domestic seaweed aquaculture. In 2024, the cultivated area in China reached 51,961 hectares, with a production of 1.86 million tons (dry weight) [3], accounting for approximately 89.40% of global *S. japonica* production. The main production regions are Fujian, Shandong, and Liaoning provinces, which contribute 50.39%, 29.13%, and 19.10% of the national yield, respectively [4].

The growth and development of *S. japonica* are closely regulated by multiple seawater environmental factors, among which salinity is one of the most important factors constraining the physiological adaptation of *S. japonica* and other large seaweeds [5,6]. Influenced by seasonal rainfall, runoff input, and tidal action, aquaculture areas often experience salinity fluctuations. Previous studies have shown that low-salinity conditions can significantly inhibit the growth and photosynthetic activity of *Saccharina latissima* and reduce the growth rate, pigment content, and Fv/Fm of its juvenile sporophytes [7]. During seedling cultivation and aquaculture of *S. japonica*, low salinity can suppress thallus growth and induce damage such as green rot at the seedling stage [8]. FAO kelp farming outcomes indicate that a sudden decrease in salinity caused by the mixing of rainwater with seawater after heavy rainfall can lead to blade decay and other damage in kelp [9]. In addition, previous studies have reported that abrupt decreases in seawater salinity, such as freshwater input caused by heavy rainfall, are important environmental factors inducing kelp blister disease and blade decay [10]. Therefore, elucidating the physiological responses and gene expression characteristics of kelp under salinity stress will not only help clarify the physiological basis underlying the development of its salinity tolerance, but also provide a reference for coping with rainfall-induced nearshore salinity fluctuations.

Existing studies have shown that salinity stress can alter the growth and physiological status of *S. japonica* and other large brown algae by affecting photosynthetic system stability, membrane lipid peroxidation, antioxidant defense, osmotic regulation, and related processes [11,12,13,14]. Under low-salinity conditions, changes in the external osmotic environment may disrupt water and ion balance in the thallus, thereby inducing reactive oxygen species (ROS) accumulation, membrane system damage, and reduced photosynthetic efficiency. To alleviate oxidative damage and osmotic imbalance caused by salinity stress, *S. japonica* and other brown algae can maintain cellular homeostasis by regulating antioxidant enzyme activities, the contents of non-enzymatic antioxidants, and the accumulation of osmolytes [13,14]. In addition, changes in the expression of some candidate genes related to antioxidant defense and osmotic regulation can also reflect the molecular responses of *S. japonica* to salinity stress. For example, glutathione S-transferase (GST), as an important component of the antioxidant defense system, and its related genes can be induced under low-salinity stress, suggesting that GST may participate in reactive oxygen species scavenging and cellular protection under salinity stress [15]. Meanwhile, the thallus of *S. japonica* exhibits pronounced tissue functional differentiation along the longitudinal axis. Different tissue regions differ in photosynthetic performance, nutrient uptake, and elemental composition [16], and different sporophyte developmental stages also show significant changes in gene expression characteristics and major metabolite composition [17]. Previous studies have further shown that different tissue regions of *S. japonica* differ in DNA methylation profiles [18], phytohormone accumulation, and the expression of related biosynthetic genes [19], photosynthetic efficiency, stress signaling pathway activity, and sulfotransferase (ST) gene expression [20,21]. These findings indicate that the response of *S. japonica* to salinity stress may depend not only on physiological processes such as photosynthesis, antioxidant defense, and osmotic regulation, but also on developmental stage and tissue functional differentiation.

Although previous studies have analyzed the stress-adaptation characteristics of *S. japonica* and closely related brown algae from the perspectives of low-salinity stress, physiological and biochemical responses, gene expression, and tissue functional differentiation, parallel comparisons among different developmental stages and tissue regions within the same salinity stress system remain limited. Existing studies have mainly focused on single aspects, such as photosynthetic parameters, antioxidant indicators, or gene expression, and comprehensive analyses integrating growth, photosynthetic damage, membrane lipid peroxidation, antioxidant defense, osmotic regulation, and candidate gene expression are still lacking. In particular, whether different tissue regions of adult-stage thalli exhibit a stable gradient of salinity tolerance, and how this gradient is associated with physiological homeostasis and the expression of stress-responsive genes, remain unclear. Therefore, in the present study, we used the new *S. japonica* strain “Haijia No. 1” as experimental material to compare the physiological responses and candidate gene expression characteristics of different developmental stages and different tissue regions of adult-stage thalli under a salinity-gradient stress regime. The null hypothesis tested in this study was that the physiological responses and candidate gene expression of *S. japonica* under salinity stress are not significantly affected by developmental stage or tissue region. By testing this hypothesis, we aimed to clarify the developmental stage-dependent differences and tissue region specificity of salinity tolerance in *S. japonica* and to provide experimental evidence for low-salinity stress risk assessment, the management of key growth stages, and the evaluation of salinity-tolerance traits.

## 2. Results

### 2.1. Differential Responses to Salinity Stress Across Developmental Stages of Saccharina japonica

**Figure 1:**
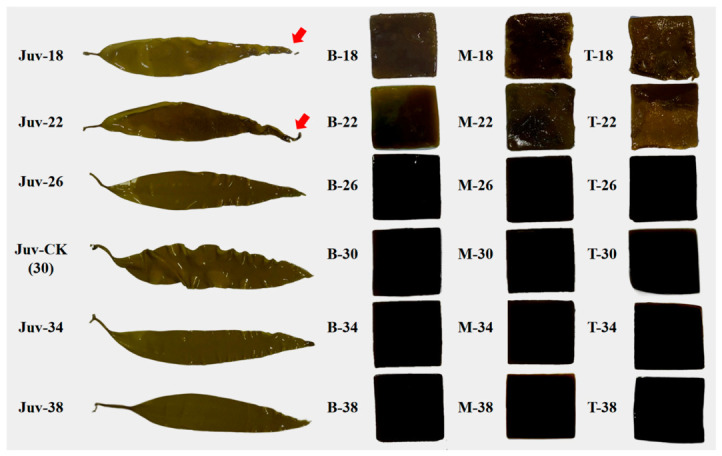
Phenotypic changes in juvenile sporophytes and adult-stage thallus tissue regions of *Saccharina japonica* after 72 h of exposure to different salinity treatments. The images on the left show intact juvenile-stage *S. japonica* thalli after 72 h of exposure to different salinity treatments. The images on the right show 3 cm × 3 cm tissue blocks excised from the basal (B), middle (M), and tip (T) regions of adult-stage thalli after 72 h of salinity stress treatment. Juv denotes juvenile-stage *S. japonica*, whereas B, M, and T denote the basal, middle, and tip regions of adult-stage thalli, respectively. The values 18, 22, 26, 30, 34, and 38 represent the salinity treatment levels, and CK denotes the control group at 30 psu. Red arrows indicate slight edge curling and discoloration in juvenile-stage *S. japonica* thalli under low-salinity stress.

The results indicate that *S. japonica* exhibited similar overall response patterns to salinity stress across developmental stages. Low-salinity treatments caused more pronounced damage than high-salinity treatments, and juvenile sporophytes generally showed higher tolerance than adult-stage thalli. Salinity stress significantly affected thallus morphology, particularly under low-salinity conditions (Figure 1). After 72 h of exposure to low salinity (18 psu and 22 psu), adult-stage thalli exhibited severe tissue damage. All regions showed varying degrees of blistering and decay, with the tip region most affected, followed by the middle region, and the basal region relatively less affected. In contrast, under high-salinity treatments (34 psu and 38 psu), the overall thallus morphology was similar to that of the control group. Juvenile sporophytes exhibited higher tolerance, showing only slight edge curling and discoloration even under low-salinity conditions.

**Figure 2:**
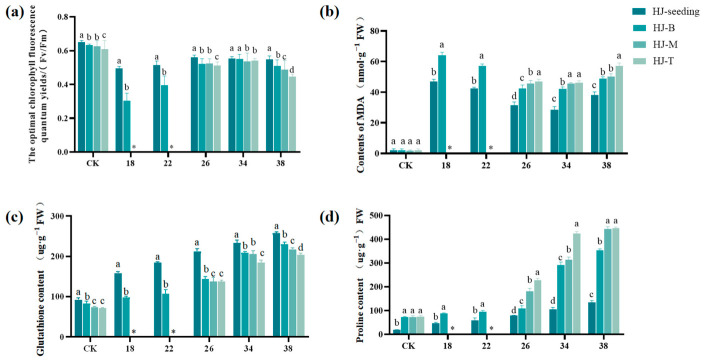
Comparison of physiological and biochemical indicators between juvenile sporophytes and adult-stage thalli of *Saccharina japonica*. (**a**) Maximum quantum yield of PSII (Fv/Fm) after 72 h of salinity stress. (**b**) Malondialdehyde (MDA) increment (72 h value minus 0 h value). (**c**) Reduced glutathione (GSH) content after 72 h of stress. (**d**) Proline (Pro) content after 72 h of stress. Juv represents juvenile sporophytes; B, M, and T represent the basal, middle, and tip regions of adult-stage thalli, respectively. The *x*-axis represents salinity levels, and CK denotes the control group at 30 psu. Different lowercase letters indicate significant differences among developmental stages or tissue regions under the same salinity treatment according to Duncan’s multiple range test (*p* < 0.05). The asterisk indicates adult-stage tissues that died or lost photosynthetic activity under 18 psu and 22 psu salinity stress after 72 h.

Physiological measurements further confirmed developmental stage-specific responses of *S. japonica* to salinity stress (Figure 2). Low-salinity stress exerted a more pronounced impact on adult-stage thalli than on juvenile sporophytes. Specifically, after 72 h of exposure to 18 psu and 22 psu salinity, juvenile sporophytes maintained relatively high maximum quantum yield of photosystem II (Fv/Fm) values, whereas Fv/Fm in the middle and tip regions of adult-stage thalli dropped to zero. Across all tissue regions, malondialdehyde (MDA) accumulation under all salinity treatments was significantly higher in adult-stage thalli than in juvenile sporophytes. Juvenile sporophytes generally exhibited higher reduced glutathione (GSH) content than adult-stage thalli across all salinity treatments, whereas proline (Pro) levels were consistently lower than those in adult-stage thalli.

### 2.2. Differential Responses of Tissue Regions in Adult-Stage Saccharina japonica to Salinity Stress

#### 2.2.1. Changes in Relative Growth Rate Under Salinity Stress

**Figure 3:**
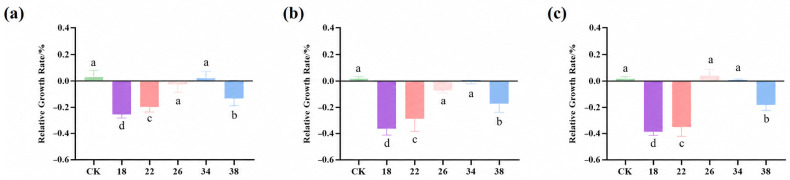
Relative growth rate (RGR) of *Saccharina japonica* under different salinity treatments. (**a**) RGR of the basal region. (**b**) RGR of the middle region. (**c**) RGR of the tip region. The *x*-axis represents salinity levels, and CK denotes the control group at 30 psu. Different lowercase letters indicate significant differences among salinity treatments within the same tissue region according to Duncan’s multiple range test (*p* < 0.05).

Under salinity stress, the relative growth rate (RGR) of the basal, middle, and tip regions in adult-stage thalli of *S. japonica* exhibited a consistent pattern (Figure 3). Specifically, the basal region exhibited the highest RGR, followed by the middle region, with the tip region showing the lowest RGR. Overall, growth inhibition was more pronounced under low-salinity conditions than under high-salinity treatments. Compared with the control, the RGR of all regions decreased most under 18 psu salinity, followed by 22 psu. High-salinity treatment (38 psu) also reduced the RGR but to a lesser extent than low-salinity stress. Within the 26–34 psu range, the RGR of all tissue regions showed minimal deviation from the control.

#### 2.2.2. Changes in Physiological Indicators Under Salinity Stress

**Figure 4:**
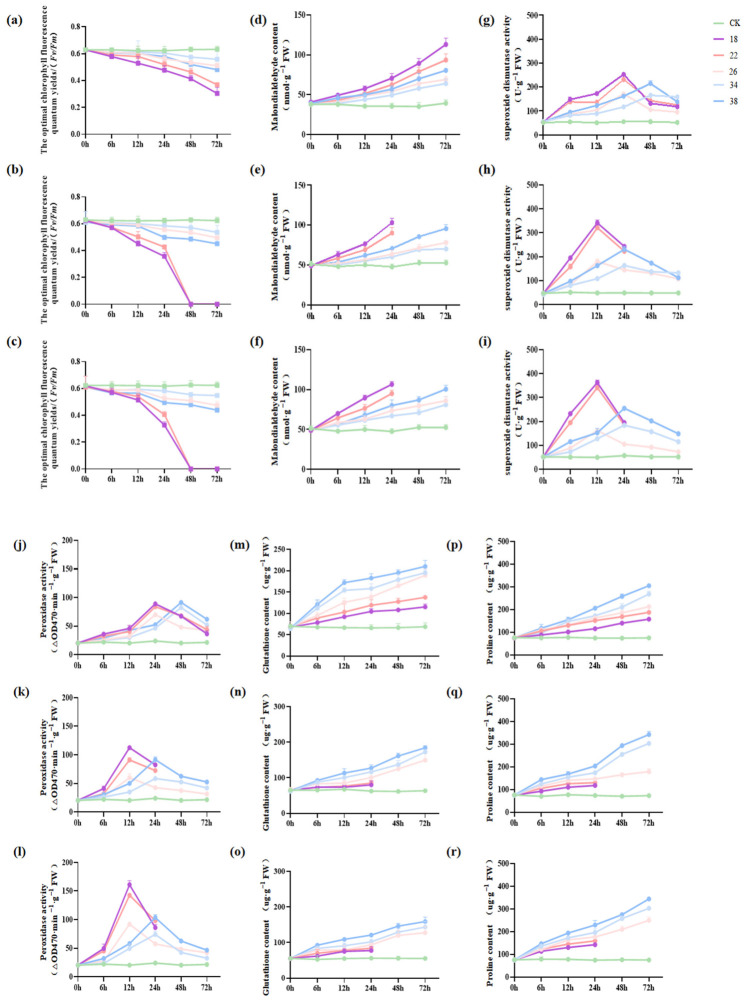
Changes in physiological indicators of different tissue regions in adult-stage thalli of *Saccharina japonica* under various salinity treatments. (**a**–**c**) Changes in Fv/Fm in the basal, middle, and tip regions, respectively. (**d**–**f**) Changes in MDA content in the basal, middle, and tip regions, respectively. (**g**–**i**) Changes in SOD activity in the basal, middle, and tip regions, respectively. (**j**–**l**) Changes in POD activity in the basal, middle, and tip regions, respectively. (**m**–**o**) Changes in GSH content in the basal, middle, and tip regions, respectively. (**p**–**r**) Changes in Pro content in the basal, middle, and tip regions, respectively. Different colors represent different salinity gradients ranging from 18 to 38 psu, and CK denotes the control group at 30 psu salinity. Data are presented as the mean ± standard deviation.

Under salinity stress, the Fv/Fm values of the basal, middle, and tip regions in adult-stage thalli of *S. japonica* showed a similar declining trend (Figure 4a–c), with the most pronounced reductions observed under 18 psu and 22 psu treatments. Specifically, under 18 psu salinity stress, Fv/Fm in the basal region declined from 0.65 at the onset of stress to 0.31 after 72 h, whereas Fv/Fm in the middle and tip regions dropped to zero at 72 h. Overall, the tolerance of tissue regions to salinity stress followed the gradient basal > middle > tip.

Regarding membrane damage, MDA content increased over time under all salinity treatments except the control, with the largest increments observed under 18 psu and 22 psu, and comparatively smaller increases under 26 psu and 34 psu treatments (Figure 4d–f). Specifically, under low-salinity stress, MDA content in the basal region continued to increase and remained high during the later stage of stress. In the middle and tip regions, MDA rapidly accumulated during the early stage and continued to rise until thallus death. Overall, MDA accumulation followed the gradient tip > middle > basal.

Under salinity stress, superoxide dismutase (SOD) and peroxidase (POD) activities in all treatment groups of *S*. *japonica* generally followed a rise-then-fall pattern, with low-salinity treatments (18 psu and 22 psu) eliciting stronger responses compared with high-salinity treatments (34 psu and 38 psu). In the basal region, activities of both enzymes peaked at 24 h under low-salinity stress, whereas the peak was delayed to 48 h under high-salinity stress. In the middle and tip regions, activities peaked at 12 h under low-salinity conditions and mostly at 24 h or later under high-salinity conditions. Overall, enzyme activity responses in the middle and tip regions were faster and more variable, whereas the basal region exhibited relatively higher stability (Figure 4g–l).

Regarding the effects of salinity stress on non-enzymatic antioxidant and osmotic regulation systems, GSH and Pro contents generally exhibited an increasing trend over time across all salinity treatments, with high-salinity treatments (34 psu and 38 psu) eliciting stronger responses than low-salinity treatments (18 psu, 22 psu, and 26 psu). GSH showed continuous accumulation in the basal, middle, and tip regions, with greater overall accumulation in the basal and middle regions than in the tip region; however, the tip region displayed a more pronounced increase during the later stages of stress. Similarly, Pro content continuously increased, with higher accumulation in the middle and tip regions than in the basal region. Collectively, both GSH and Pro participated in the response of *S. japonica* to salinity stress, but clear tissue-specific differences were evident in non-enzymatic antioxidant and osmotic regulation responses (Figure 4m–r).

### 2.3. Effects of Salinity Stress on the Expression of Antioxidant Enzyme-Related Genes in Saccharina japonica

**Figure 5:**
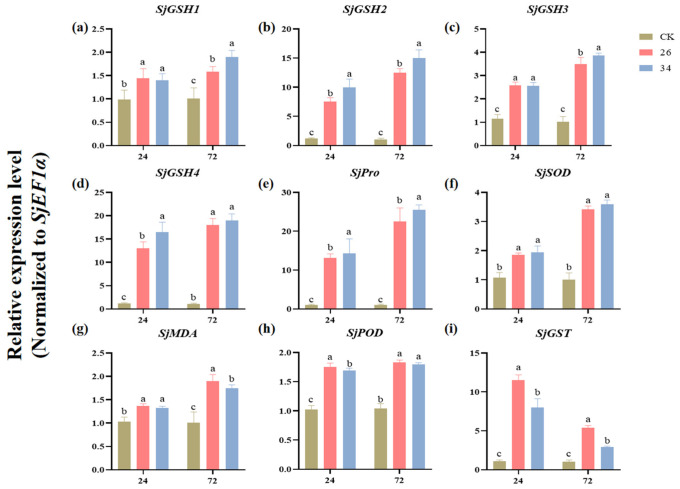
Effects of salinity stress on the expression of antioxidant enzyme-related genes in *Saccharina japonica*: *SjGSH1* (**a**), *SjGSH2* (**b**), *SjGSH3* (**c**), *SjGSH4* (**d**), *SjPro* (**e**), *SjSOD* (**f**), *SjMDA* (**g**), *SjPOD* (**h**), and *SjGST* (**i**). Expression levels were normalized to *SjEF1α* as the reference gene. The *x*-axis represents sampling time points at 24 h and 72 h. Data are presented as the mean ± standard deviation. CK represents the control group at 30 psu salinity, while 26 and 34 represent salinity treatments at 26 psu and 34 psu, respectively. Different letters indicate significant differences among salinity treatments at the same time point according to Duncan’s multiple range test (*p* < 0.05).

To investigate the transcriptional regulation of key antioxidant and stress-responsive genes under salinity stress, we analyzed the expression of multiple antioxidant enzyme-related genes in adult-stage thalli of *S. japonica* using quantitative real-time PCR (qRT-PCR). The results indicate that all examined antioxidant enzyme-related genes were significantly upregulated under salinity stress (Figure 5). At 24 h under 26 psu salinity, the expression levels of *SjGSH1*, *SjGSH2*, *SjGSH3*, *SjGSH4*, *SjPro*, *SjSOD*, *SjMDA*, *SjPOD*, and *SjGST* were significantly upregulated to varying degrees, indicating that these genes may be involved in the response of *S. japonica* to salinity stress. By 72 h, except for *SjGST*, whose expression level decreased markedly compared with that at 24 h, the remaining candidate genes generally maintained relatively high expression levels under the 26 psu and 34 psu treatments.

## 3. Discussion

### 3.1. Salt Tolerance Differences Across Developmental Stages of Saccharina japonica

Previous studies have found that, under low-salinity stress, the growth, pigment accumulation, and Fv/Fm of juvenile sporophytes of *S. japonica* are inhibited, indicating that the photosynthetic system and basal metabolism of *S. japonica* are sensitive to low-salinity stress [11]. Studies on adult *S. japonica* have also confirmed that salinity changes can significantly affect PSII photochemical efficiency [12]. Similar findings have been reported in the European sugar kelp *Saccharina latissima*, in which low-salinity stress inhibited kelp growth and the photosynthetic system while inducing physiological and biochemical responses such as osmolyte accumulation and antioxidant defense [22,23]. Together, these studies suggest that kelp is sensitive to salinity changes at different developmental stages, including juvenile sporophytes and adult thalli. Moreover, differences among developmental stages in photosynthetic responses, basal metabolism, and stress-regulation capacity indicate that salinity tolerance in kelp may exhibit a pronounced developmental stage dependence.

In the comparison between juvenile sporophytes and adult-stage thalli, we further found that juvenile thalli maintained relatively high Fv/Fm under low-salinity conditions and exhibited lower MDA accumulation and higher GSH levels, indicating that their stronger salinity tolerance may mainly depend on a more stable photosynthetic system and stronger basal antioxidant capacity. An increase in MDA content usually indicates intensified membrane lipid peroxidation and cell membrane system damage [24]. As an important non-enzymatic antioxidant, GSH participates in ROS scavenging and the maintenance of redox homeostasis [25]. Previous studies on stress responses in large seaweed have shown that salinity stress can induce ROS accumulation, leading to increased MDA levels, reduced cell membrane integrity, and changes in the antioxidant system [26,27,28]. In contrast, adult-stage thalli were more sensitive to low-salinity stress, which may be associated with their higher degree of thallus tissue differentiation and more pronounced physiological functional differences among tissue regions. Under rapid changes in the external osmotic environment caused by low salinity, adult-stage thalli may be more prone to water and ion imbalance, thereby inducing ROS accumulation, aggravated membrane lipid peroxidation, and photosynthetic system inactivation. It is worth noting that the Pro content in juvenile thalli was lower than that in adult-stage thalli in this study. Pro usually acts as an osmolyte and participates in the regulation of cellular osmotic potential, the stabilization of proteins and membrane structures, and the buffering of oxidative damage [29]. In a study on annual *Medicago* spp., it was found that high Pro content was associated with sensitivity to salt stress and suggested that Pro biosynthesis may be a consequence of disturbed cellular homeostasis, reflecting poorer growth performance and more severe stress injury [30]. Based on the results of the present study, the higher Pro accumulation in adult-stage thalli may represent a compensatory osmotic regulatory response under low-salinity stress, rather than necessarily indicating stronger salinity tolerance.

### 3.2. Tissue-Specific Responses to Salinity Stress

Previous studies have shown that the thallus of *S. japonica* exhibits functional differentiation among different tissues along the growth axis [31]. In their study of different generations and thallus regions of *S. japonica*, Wang et al. also found that different regions differed in PSII photochemical efficiency, nutrient uptake efficiency, and C and N elemental composition, with the basal region showing higher C content and C/N ratio [16]. A developmental transcriptomic analysis of *S. japonica* further indicated that the basal blade was more closely associated with processes related to meristematic growth, cell division, and carbon metabolism [32]. These studies suggest that the basal, middle, and tip regions of *S. japonica* are not functionally homogeneous tissues, but instead exhibit intrinsic differences in photosynthetic performance, resource accumulation, cell-wall-related processes, and developmental status. Based on this tissue functional differentiation, the present study further demonstrated that different tissue regions of adult-stage thalli differed markedly in their tolerance to salinity stress. This tolerance gradient, with stronger tolerance in the basal region and weaker tolerance in the tip region, may be associated with differences in developmental status and physiological functional partitioning along the longitudinal axis of the thallus.

On the basis of the above tissue functional differentiation, the differences in physiological responses among tissue regions under salinity stress may be further associated with differences in tissue age, photosynthetic activity, and ROS-scavenging capacity. Previous studies have shown that, under salt stress, the apical or more mature regions of plant leaves are generally more prone to ROS accumulation, membrane lipid peroxidation, and chloroplast structural damage, whereas basal or younger tissues usually experience relatively mild oxidative damage due to their stronger ROS-scavenging capacity [33,34,35]. Studies on the large brown alga *Macrocystis pyrifera* have also shown that apical and basal blades exhibit differential photosynthetic responses under complex environmental gradients characterized by low salinity, low temperature, and high turbidity caused by glacial meltwater influence [36]. Consistent with these findings, the higher MDA accumulation and more pronounced phenotypic damage observed in the tip region in the present study suggest that this region may experience stronger photoinhibition and membrane lipid peroxidation pressure under low-salinity stress. In contrast, the lower level of membrane lipid peroxidation and the relatively moderate changes in antioxidant enzyme activities in the basal region indicate that this region may have a more stable capacity for ROS scavenging and the maintenance of cellular homeostasis. In addition, the higher Pro accumulation in the middle and tip regions may reflect stronger osmotic regulatory responses activated in these regions under low-salinity stress. However, this response is more likely to represent a compensatory defense after damage has occurred and may not be sufficient to fully prevent photosynthetic system inactivation and tissue injury. Therefore, the differences in salinity tolerance among tissue regions of adult-stage thalli are not the result of changes in a single physiological indicator but are likely caused by the combined effects of tissue functional differentiation, the degree of oxidative damage, antioxidant defense capacity, and osmotic regulatory responses.

### 3.3. Expression Responses of Candidate Genes Related to Antioxidant Defense and Osmotic Regulation in Saccharina japonica Under Salinity Stress

Previous studies have shown that abiotic stresses such as salinity and high temperature can induce oxidative damage and osmotic imbalance in *S. japonica*, and that cellular homeostasis can be maintained by regulating antioxidant enzyme activities, glutathione metabolism, related gene expression, and osmolyte accumulation [37,38,39,40]. Therefore, antioxidant defense and osmotic regulation may constitute important physiological bases for the response of *S. japonica* to environmental stress. Based on the physiological responses described above, the qRT-PCR results of the present study further show that candidate genes such as *SjSOD*, *SjPOD*, *SjPro*, *SjGSH,* and *SjGST* exhibited different degrees of expression changes after salinity treatment, indicating that the response of *S. japonica* to salinity stress was accompanied by transcriptional regulation of pathways related to antioxidant defense, glutathione metabolism, and osmotic regulation.

A study by Cui et al. on high-temperature stress in *S. japonica* showed that the activities of protective enzymes such as SOD and POD and the expression of antioxidant-related genes were induced by stress, while Pro content also increased significantly, suggesting that antioxidant defense and osmotic regulation may represent common mechanisms by which *S. japonica* responds to different abiotic stresses [38]. In the present study, *SjSOD* and *SjPOD* were significantly upregulated at the measured 24 h time point under salinity stress, and their expression patterns were generally consistent with changes in SOD and POD enzyme activities, which is consistent with the findings of Cui et al. *SjSOD* can catalyze the dismutation of superoxide anion (O_2_^−^) to generate H_2_O_2_ [41], whereas *SjPOD* further participates in H_2_O_2_ scavenging, thereby helping to alleviate membrane lipid peroxidation damage caused by ROS accumulation [42]. However, MDA content still increased with increasing stress intensity in the present study, indicating that the activation of antioxidant-related genes and enzyme activities may not fully offset the oxidative damage caused by sustained salinity stress. With respect to osmotic regulation, the upregulation of *SjPro* in this study corresponded with the trend of Pro accumulation, indicating that Pro-related metabolic pathways were induced under salinity stress. This is generally consistent with previous findings that abiotic stress can promote Pro accumulation [38,43]. However, when considered together with the developmental stage-dependent and tissue region-specific differences observed in this study, Pro accumulation is more likely to reflect a compensatory osmotic regulatory response following the disturbance of cellular homeostasis under salinity stress.

In addition, changes in the expression of *SjGSH1–4* and *SjGST* further indicate that glutathione-related antioxidant and detoxification pathways participate in *S. japonica*’s response to salinity stress. GST can use GSH as a substrate to participate in the removal of membrane lipid peroxidation products and in cellular detoxification [44]. Previous studies on the GST gene family in *S. japonica* have shown that most *SjGST* genes can be induced by low-salinity stress [15]. Consistent with these findings, *SjGST* was significantly upregulated at 24 h in the present study, suggesting that the GST-mediated glutathione detoxification pathway may be involved in the response detected at 24 h under salinity stress. Notably, the expression level of *SjGST* decreased markedly at 72 h, whereas most *SjGSH* genes and other candidate genes maintained relatively high expression levels. This suggests that different temporal response patterns may exist within glutathione-related pathways: *SjGST* may contribute more strongly to the glutathione detoxification response detected at 24 h, whereas *SjGSH*-related genes may contribute to glutathione metabolism and the maintenance of redox homeostasis at 72 h.

## 4. Conclusions

In this study, we used the new *S. japonica* strain “Haijia No. 1” as experimental material and systematically analyzed the physiological responses and candidate gene expression characteristics of *S. japonica* under salinity stress at different developmental stages, namely, the juvenile and adult stages, and in different tissue regions of adult-stage thalli, namely, the basal, middle, and tip regions. The results show that low-salinity stress caused more severe damage to *S. japonica* than high-salinity stress, mainly manifested as a decline in the maximum photochemical efficiency of photosystem II (Fv/Fm), aggravated membrane lipid peroxidation, and increased tissue damage. Salinity stress tolerance differed among developmental stages, with juvenile thalli showing higher overall tolerance than adult-stage thalli. Different tissue regions of adult-stage thalli exhibited clear spatial differences. The basal region maintained a relatively high relative growth rate and photosynthetic activity under salinity stress and showed lower membrane lipid peroxidation and relatively stable antioxidant and osmotic regulatory responses. In contrast, the tip region was more sensitive to low-salinity stress and more prone to photosynthetic activity loss and tissue damage. The qRT-PCR results further showed that candidate genes, including *SjGSH*, *SjGST*, *SjPro*, *SjSOD*, and *SjPOD*, exhibited significant expression responses under salinity stress, suggesting that antioxidant defense and osmotic regulation may participate in the adaptation of *S. japonica* to salinity changes. Overall, this study clarified the developmental stage-dependent and tissue region-specific differences in the salinity stress response of *S. japonica*, indicating that the tip region of adult-stage thalli may serve as a sensitive region for low-salinity damage, whereas the basal region may be an important region for maintaining thallus growth and physiological homeostasis. These results provide experimental evidence for low-salinity stress risk assessment, the management of key growth stages, the monitoring of sensitive tissues, and the evaluation of salinity-tolerance traits during *S. japonica* aquaculture.

## 5. Materials and Methods

The new *S. japonica* cultivar “Haijia No. 1” was used as an experimental material. Samples were collected from the Xiapu coastal area, Fujian Province, China (26°32′59.02″ N, 119°57′7.37″ E), and then maintained in the laboratory for 3 days until their physiological status stabilized. Salinity stress experiments were subsequently conducted separately using juvenile and adult-stage thalli. Adult-stage thalli were further divided into basal (B), middle (M), and tip (T) regions to compare region-specific responses to salinity stress.

Intact juvenile and adult-stage thalli were selected from the collected samples and transferred to 1 L glass aquaria. Culture conditions were as follows: a light intensity of 40–50 μmol photons/(m^2^·s), a temperature of 12 °C, a 12 h light/12 h dark photoperiod, and sterile natural seawater as the culture medium. Nutrients were supplied as NaNO_3_ (4 mg/L NO_3_^−^–N; Sinopharm Chemical Reagent Co., Ltd., Shanghai, China) and KH_2_PO_4_ (0.4 mg/L PO_4_–P; Sinopharm Chemical Reagent Co., Ltd., Shanghai, China), which provided nitrogen and phosphorus, respectively. Cultures were maintained in a light incubator (AL-200-3, Wuhan Ruihua Instrument Equipment Co., Ltd., Wuhan, China) under aerated suspension conditions. For adult-stage thalli, 3 cm × 3 cm tissue blocks were excised from the basal, middle, and tip regions of each independent thallus, and samples from different tissue regions were cultured separately.

### 5.1. Experimental Design

For the experiment, we used filtered natural seawater as the base culture medium, with 30 psu salinity set as the control. Six salinity treatments were established: control (30 psu), low-salinity stress (18 psu, 22 psu, and 26 psu), and high-salinity stress (34 psu and 38 psu). Four independent biological replicates were established for each salinity treatment. For juvenile-stage *S. japonica*, each biological replicate consisted of one independent juvenile-stage thallus. For adult-stage thalli, each biological replicate consisted of one independent adult-stage thallus, from which 3 cm × 3 cm tissue blocks were excised from the basal, middle, and tip regions.

Salinity stress was applied for 72 h, and samples were collected at 0, 6, 12, 24, 48, and 72 h for subsequent physiological, biochemical, and molecular analyses. At 72 h, the thalli of juvenile sporophytes and the basal (B), middle (M), and tip (T) regions of adult-stage thalli were photographed to observe phenotypic changes. Samples were labeled according to salinity and tissue region; for example, Juv-18 represents juvenile sporophytes under 18 psu salinity stress, and B-18, M-18, and T-18 represent the basal, middle, and tip regions of adult-stage thalli under 18 psu salinity stress; other salinity treatments followed the same labeling convention.

All salinity treatment solutions were prepared using natural seawater as the base. The initial salinity of the seawater was measured and adjusted to 30 psu for the control group. Low-salinity treatments were prepared by gradually diluting natural seawater with distilled water to obtain final salinities of 26 psu, 22 psu, and 18 psu, while high-salinity treatments were prepared by gradually adding NaCl to obtain final salinities of 34 psu and 38 psu. After each addition of distilled water or NaCl, the solution was thoroughly mixed, and salinity was remeasured until the target value was reached. To minimize preparation errors, all treatment solutions were prepared in advance, and salinity was reconfirmed before use. During the experiment, the culture volume was kept consistent across all treatments so that salinity was the only variable. The specific methods used for the determination of each physiological parameter are described below.

(1) Determination of RGR: To compare the growth responses of *S. japonica* under different salinity treatments, RGR was used to characterize growth changes during the stress period. Before the salinity treatment began, the initial fresh weight of each sample was measured and recorded as W_1_; at the end of the treatment, the final fresh weight was measured and recorded as W_2_. For each measurement, the sample was removed from the culture medium, surface moisture was gently blotted off with absorbent paper, and the sample was weighed immediately to ensure the accuracy of fresh weight determination. Juvenile-stage samples were measured as whole thalli, whereas adult-stage thalli samples were measured separately for the basal, middle, and tip tissue sections. The starting and ending times of the treatment were recorded as t_1_ and t_2_, respectively. RGR was calculated using the following equation:

RGR = (ln W_2_ − ln W_1_)/(t_2_ − t_1_)

where W_1_ and W_2_ represent the fresh weights (g) of the samples at the beginning and end of the treatment, respectively, and t_2_ − t_1_ represents the treatment duration (d).

(2) Determination of chlorophyll fluorescence parameters: To investigate changes in photosystem II function in the thalli under salinity stress, chlorophyll fluorescence parameters of *S. japonica* samples were measured using a DUAL-PAM-100 chlorophyll fluorometer (WALZ, Bavaria, Germany). Before measurement, the samples were removed from the culture medium, and surface-adhering water was gently blotted with absorbent paper. The thalli were then dark-adapted for 20 min, after which the minimum fluorescence (Fo) and maximum fluorescence (Fm) under dark-adapted conditions were measured. The maximum quantum yield of photosystem II was calculated using the formula Fv/Fm = (Fm − Fo)/Fm, where Fv represents variable fluorescence. For both juvenile-stage and adult-stage samples, measurements were taken from the central area of each tissue block, while visibly damaged areas were avoided to reduce the effects of tissue heterogeneity and local damage on the measurement results.

The maximum quantum yield of photosystem II (Fv/Fm) was calculated from the dark-adapted minimum fluorescence (Fo) and maximum fluorescence (Fm), where Fv = Fm − Fo. In this study, Fv/Fm was used as a chlorophyll fluorescence indicator to evaluate the potential photochemical efficiency of photosystem II and the degree of photosynthetic damage in *S. japonica* under salinity stress.

(3) Determination of physiological parameters: To evaluate the degree of oxidative damage, antioxidant defense capacity, and osmotic adjustment responses of *S. japonica* at different developmental stages and in different tissue regions under salinity stress, the contents of MDA, reduced GSH, Pro, and SP, as well as the activities of SOD and POD, were determined. Except for soluble protein, which was measured using a kit purchased from Ruixin Biotechnology Co., Ltd. (Quanzhou, China), all other parameters were determined using assay kits purchased from Beijing Solarbio Science & Technology Co., Ltd. (Beijing, China). Approximately 0.1 g of *S. japonica* tissue was used for each assay. For adult-stage thalli samples, the middle area of each tissue section was uniformly selected for measurement, and visibly damaged regions were avoided to minimize the effects of tissue heterogeneity and local necrosis on the results. Samples were homogenized in extraction solution in an ice bath and centrifuged at 4 °C, after which the supernatants were collected for analysis according to the manufacturers’ instructions. The absorbance values of MDA, SOD, POD, GSH, and Pro were measured at 532/600, 450, 470, 412, and 520 nm, respectively, and the corresponding contents or enzyme activities were then calculated.

### 5.2. RNA Extraction and Real-Time Fluorescent Quantitative Analysis

Total RNA was extracted from the *S. japonica* samples using a total RNA extraction kit (Omega Bio-tek, Inc., Norcross, GA, USA), and first-strand cDNA was synthesized using the TransScript All-in-One First-Strand cDNA Synthesis SuperMix kit (one-step, with gDNA removal; TransGen Biotech Co., Ltd., Beijing, China). The synthesized cDNA was diluted threefold and used as the template for quantitative real-time PCR (qRT-PCR).

qRT-PCR reactions were performed using PerfectStart Green qPCR SuperMix reagent (TransGen Biotech Co., Ltd., Beijing, China). Each reaction was carried out in a total volume of 20 μL containing 10 μL of 2 × PerfectStart Green qPCR SuperMix, 0.4 μL of forward primer (10 μM), 0.4 μL of reverse primer (10 μM), 1 μL of diluted cDNA template, and 8.2 μL of nuclease-free water. The qRT-PCR amplification program was as follows: initial denaturation at 94 °C for 30 s, followed by 40 cycles of denaturation at 94 °C for 5 s and annealing/extension at 60 °C for 30 s. After amplification, melting curve analysis was performed to verify the specificity of the amplified products. Three independent biological replicates were set for each treatment, and three technical replicates were performed for each biological replicate. No-template controls were included to exclude contamination of the reaction system, and no-reverse-transcription controls were included to exclude interference from residual genomic DNA.

Based on the selection of reference genes in previous qRT-PCR studies of *S. japonica*, *SjEF1α* was used as the reference gene in this study to normalize the expression levels of target genes. The relative expression levels of each candidate gene were calculated using the 2^−ΔΔCt^ method [45]. Target gene sequences were obtained from the *S. japonica* genome database, and specific primers were designed using Primer Premier 5.0 software (Premier Biosoft International, Palo Alto, CA, USA). The primer sequences are listed in Table 1.

### 5.3. Data Analysis

Data were collected and preliminarily processed using Excel 2021. Statistical analyses were performed using IBM SPSS Statistics 27 software (IBM Corporation, Armonk, NY, USA). To evaluate significant differences in photosynthetic parameters and physiological indices among different treatment groups, one-way analysis of variance (ANOVA) was conducted, followed by Duncan’s multiple range test for multiple comparisons among groups. Graphs and figures were generated using GraphPad Prism 9 software (GraphPad Software, San Diego, CA, USA). All data are presented as mean ± standard deviation (Mean ± SD).

## Figures and Tables

**Table 1:** Gene primer sequences used for quantitative real-time PCR analysis.

Gene	Forward Primer (5′–3′)	Reverse Primer (5′–3′)
*SjEF1α*	GTGATGGAGGAGAACCC	TTGATGACACCCACAGC
*SjGSH1*	GACAGCCTCGTCCTTATCG	TCGTTGCCGCCGTAGTAT
*SjGSH2*	CGGCAATGGTCTAGCTTGGA	CCTCGGCGTACTTGTTGGT
*SjGSH3*	AGCACTTGAAGGAGCAGATGG	CGCCTACGGTGGTGTTGAA
*SjGSH4*	CCATTGCTGACTGCTGACTATC	GCTGGTGAGTTGGCTTGTG
*SjPro*	AGCCGACGAAGAACGAGATT	GTCCTCCTCCGTCATCACAC
*SjSOD*	CCACTACAACCACTGCCTCTT	CGCCGAACTGCTCCTTCAT
*SjMDA*	ATGGCGAAGTATGATGAGTTGT	AAGGACAGGAACATGACGAATC
*SjPOD*	TCGGAGATGAGGGGATCGTT	GCTGTTGTCGAACTTGAGCC
*SjGST*	AGAAGGACGAGACCAAGAAGT	GTAGGCGGAGCACTTGTTG

## Data Availability

Data are available on request from the corresponding authors.
